# Investigation of Physicho-chemical Properties and Characterization of Produced Biosurfactant by Selected Indigenous Oil-degrading Bacterium

**Published:** 2018-08

**Authors:** Ziba NAJMI, Gholamhossein EBRAHIMIPOUR, Andrea FRANZETTI, Ibrahim Mohamed BANAT

**Affiliations:** 1. Dept. of Microbiology, Faculty of Life Sciences and Biotechnology, University of Shahid Beheshti, Tehran, Iran; 2. Dept. of Earth and Environmental Sciences, University of Milano-Bicocca, Milan, Italy; 3. School of Biomedical Sciences, Faculty of Life and Health Sciences, University of Ulster, Coleraine, UK

**Keywords:** Screening, *Pseudomonas aeruginosa*, Characterization, Rhamnolipids

## Abstract

**Background:**

Due to the amphipathic properties of biosurfactants which act on surfaces and interfaces interest by a variety of industries such as cosmetic, pharmaceutical, bioremediation and petroleum-related industries has recently increased.

**Methods:**

Detection of a high-efficiency biosurfactant producer using preliminary screening methods from soil contaminated with crude oil was carried at the Microbiology Laboratory at Shahid Beheshti University, Tehran, Iran in 2013. Then after characterization of some physico-chemical properties of produced biosurfactant and production optimization conditions, processes of purification and complete identification were done.

**Results:**

*Pseudomonas aeruginosa* sp. ZN was selected as high-efficiency biosurfactant producing strain from soil contaminated with oil from Ahvaz City, Khuzestan Province, southern Iran. The biosurfactant production in modified BH2 culture medium supplemented with 1% n-hexadecane occurred during exponential phase resulting in a reduction surface tension from 70 to 29 mN/m. Strain ZN produced biosurfactant with different properties to other *Pseudomonas* reported. These characterizations included continued production at C/N ratio range of 10–40; the produced biosurfactant could not separate stable emulsion of span-80-kerosene: Tween-80-distilled water (30:70) within 24 h. The produced biosurfactants were able to increase hydrophobicity of bacterial cell to 55%. Recovery of biosurfactants from cell-free supernatant was performed with acid precipitation and ammonium sulfate precipitation. Chemical analysis such as spraying techniques on developed TLC plate and staining methods of supernatant indicated that produced biosurfactants were glycolipids, characterized by ESI-MS analysis of extracted product as di-rhamnolipids.

**Conclusion:**

Ability of this strain to produce biosurfactant in the presence of cooked oil and n-hexadecane make it an optimistic candidate for biodegradation of some derivatives of crude oil and food industry.

## Introduction

Biosurfactants are either secreted extracellularly or attached to cell-bound compounds that decrease surface and interfacial tension and are produced by a large number of prokaryotic and eukaryotic microorganisms (1). Biosurfactants are categorized into lipoproteins, lipopolysaccharides, phospholipids, glycerides, and glycolipids according to their chemical groups and microbial origins (2, 3).

Interest in use of biosurfactants in different industries such as bioremediation, biodegradation, petroleum, medicine, cosmetic and food industries, increased in recent years due to their advantages over chemical surfactants including biodegradability, lower toxicity, better environmental compatibility and specific activity at extreme temperatures, salinities and pHs (1, 4).

The composition of culture media and chemo-physical parameters strongly influence kinetic production, biosurfactant congeners and properties (5, 6). The amphiphilic nature of biosurfactants not only form uniform water in oil and oil in water emulsions but could also dehydrate emulsions, being a promising technique in petroleum dependent industries (7).

The purpose of this study is isolation of a high-efficiency biosurfactant producing strain among hydrocarbon degraders and complete identification of produced biosurfactant based on purification, preliminary characterization and ESI-MS results.

## Materials and Methods

### Bacterial Isolates

The 10 mg of soil contaminated with crude oil collected from Ahvaz City, Khuzestan Province, southern Iran in 2013, was suspended in 100 ml buffer (solution A in modified Bushnell-Haas2 (BH2) medium), after shaking at 130 rpm, 5 ml of this suspension was added to modified BH2 medium supplemented with 1% (v/v) crude oil. For re-inoculation, after 20 d, 1 ml of fermentation broth transferred to fresh medium. Finally, 100 μl culture broth steaked on nutrient agar and incubated at 30 °C for 24 h.

### Culture media conditions and isolation of high-efficiency producing bacterium

The compositions of nutrient broth for preparing of inoculum were as follows (g/L): beef extract 1.0, yeast extract 2.0 and peptone 5.0 in distilled water. For screening biosurfactant producers a Mineral Salt Medium (MSM) with following compositions (g/L) was utilized: KH_2_PO_4_, 0.5; NH_4_Cl, 1; MgSO_4_·7H_2_O, 0.2; FeSO_4_·7H_2_O, 0.01; CaCl_2_·2H_2_O, 0.01 and 1ml of trace element solution containing (mg/L): ZnCl_2_, 70; MnCl_2_·4H_2_O, 100; CoCl_2_·6H_2_O, 200; NiCl_2_·6H_2_O, 100; CuCl_2_·2H_2_O, 20; NaVO_3_·H_2_O, 10; Na_2_WO_4_·2H_2_O, 30; Na_2_SeO_3_·5H_2_O, 26; NaMoO_4_·2H_2_O, 50 and HCl 0.25%, 1 ml and also 1% (v/v) crude oil as carbon source. To determine optimization conditions for biosurfactant production, modified BH2 medium containing with solution A (g/L): K_2_HPO_4_·3H_2_O, 1.73; KH_2_PO_4_, 1; NH_4_Cl, 0.81; NaNO_3_, 0.84; and solution B (g/L): MgSO_4_·7H_2_O, 1M; FeSO_4_·7H_2_O, 0.01; CaCl_2_·2H_2_O, 1M was used. Cultivations were performed in 500 ml flasks containing 100 ml culture media with pH 7, and stirred in a rotary shaker at 130 rpm and 30 °C for 10 d.

Fermentation broths were centrifuged at 5000 rpm at 4 °C for 10 min to remove bacterial cells. To detect biosurfactant producers some preliminary screening methods were performed (8–10).

### 16S rDNA gene sequence analysis

For partial 16S rDNA sequence analysis, following isolation bacterium chromosomal DNA with High Pure PCR template preparation kit (Roche Applied Science, Germany), polymerase chain reaction (PCR) was carried out using CinnaGen PCR Master Mix (Cat No. PR8252C) containing MgCl_2_, PCR buffer, dNTP mixtures and enzymes. Afterward, primers and template DNA were added. In this study, one set of primer 27f (forward primer 5′-AGAGTTTGATCCTGGCTCAG) / 1510r (reverse primer 5′-TACGGYTACCTTGTTA CGACTT) was used to amplify conserved sequences of the bacterial 16S rDNA. The final volume of reaction was 50 μl including 25 μl Master Mix, 1 μl of each primer, 1 μl template DNA and the rest of volume, sterile deionized water was added. PCR amplification was carried out using a thermal cycler (Techne, UK) with following program: initial denaturation at 94 °C for 5 min, then 33 cycles of denaturation at 94 °C for 30 sec, primer annealing at 56 °C for 55 sec and primer extension at 72 °C for 1 min. The PCR product was sequenced by CinnaClone Company and sequence homologies were performed with NCBI. A consensus neighbor-joining tree was constructed using Molecular Evolutionary Genetics Analysis (MEGA) software version 5.0. The sequencing result was submitted to GenBank with accession number KX808675.

### Kinetic of biosurfactant production and measurement of surface tension and CMD

Culture medium (modified BH2) supplemented with 1% (w/v) n-hexadecane and initial OD_600_ of 0.1 used to produce biosurfactant for 6-day incubation. At regular intervals, 10 ml of sample was taken for determination of OD_600_, surface tension (ST) and critical micelle dilution (CMD) at room temperature with De Nouy ring method using K-8 tensiometer (Kruss, Hamburg, Germany). Critical micelles dilution (CMD) is a measure of the dilution factor to reach the level of critical micelle concentration (1).

### Effect of carbon sources and C/N ratios on biosurfactants production

In order to determine the optimum conditions for biosurfactant production, modified BH2 culture medium with different carbon sources such as: decane, n-hexadecane, glucose, glycerol, paraffin and cooked oil, at concentration of 1% (w/v) and after selection the best carbon source, different C/N ratios such as: 10, 20, 25, 30, 35 and 40 were used. The culture media were incubated at 30 °C, pH 7 and on rotary shaker at 130 rpm for 10 d. The biosurfactant production and its activity were investigated oil spreading test and E24, respectively at regular time intervals.

### Emulsification Index (E24)

Two ml of paraffin was added to the same volume of cell-free supernatant in a glass test tube. The tube was mixed vigorously for 2 min and then left at room temperature for 24 h. E24 was calculated as (h_1_/h_0_) × 100; where h_1_ is the height of emulsified layer (mm) after 24 h and h_0_ is the total height of liquid column (mm) (11).

### Bacterial adhesion to hydrocarbon test (BATH test)

BATH test is indicative of bacterial cell hydro-phobicity in the presence of biosurfactant. One ml of fermentation broth was transferred to a microcentrifuge tube and centrifuged to separate bacterial cells. Then, the cell pellets were washed and suspended in buffer part of modified BH2 medium to obtained approximately 1 for OD_600_ and measures it as A_i_. 200 μl of hydrocarbon (nhexadecane or n-heptadecane) was added and vortexed severely for 2 min. After 30 min under steady condition, OD_600_ (A_f_) of the aqueous phase was measured and cell hydrophobicity (CH %) was calculated as (1-A_f_/A_i_) × 100 (5, 12).

### Antimicrobial activity

Antimicrobial activity was investigated against *Escherichia coli* as a gram-negative bacterium, *Bacillus subtilis* as a gram-positive bacterium and *Candida albicans* as yeast with disk diffusion method. After incubation time for 24 h at 37 °C, diameter of inhibition zones was measured (12,13).

### Demulsification experiment

For preparing the stock solutions of kerosene and Tween-80, 0.8 g of Span-80 (Sigma, Lot No. 98k09888) and 1 g of Tween-80 were added to 1 L of kerosene and 1 L of distilled water, respectively. Before using, they have been stirred for 1 min. To identify model emulsions, different ratios of Span-80-kerosene: Tween-80-distilled water (70:30, 60:40, 50:50, 40:60 and 30:70) were performed. The total volume was 10 ml. Then, they were mixed on a vortex at maximum speed for 3 min and were incubated at 30 °C under a static condition. After 24 h, the most stable emulsion was selected as a model emulsion. To carry out demulsification assay, 1 ml of cell-free supernatant was added to 9 ml of model emulsion and mixed vigorously 3 min to form a uniform emulsion and incubated for 24 h (7).

### Recovery and purification of biosurfactants

Two methods were carried out to recover biosurfactants: acid precipitation and ammonium sulfate precipitation. For acid precipitation method, pH of cell-free supernatant was decreased to below 2 with HCl or H_2_SO_4_ and followed by storing at 4 °C for overnight. The pellet contained biosurfactants were removed by centrifugation (10000 × g, 20 min) and dissolved in sodium bicarbonate (pH 8.6), in the next step, acidification was carried out again (5). In order to carry out ammonium sulfate precipitation, 40% of this compound was added to cell-free supernatant and incubated overnight at room temperature. The floating materials were collected by centrifugation (10000 × g, 20 min) and dissolved in distilled water (14).

For further extraction, the fraction contained biosurfactant was washed by the same volume of ethyl acetate three times (15).

Although a large volume of impurities was removed in the former recovery steps, there were some residual hydrocarbons and bacterial metabolites which co-extracted with biosurfactant. To address this problem and also separation of different biosurfactant congeners, liquid column chromatography was an advantageous technique. A 26 × 3.3 cm column filled up with 20 g silica gel 60 (200–425 mesh)-ethyl acetate slurry and then loaded with 1 g of biosurfactant in ethyl acetate. To remove hydrocarbons used as carbon sources, initially the column was washed with ethyl acetate and after that washed with 40 ml of different ratios of ethyl acetate: methanol (100, 90:10, 60:40 and 100). Oil spreading test was carried out for each fraction (16).

### Preliminary biosurfactant characterization

Partially purified biosurfactant with acid precipitation and column chromatography was used for all process of biosurfactant characterization. The primary biosurfactant structures were determined with a variety of spraying techniques on thin layer chromatography (TLC). In this method, the developing solvent was ethyl acetate: hexane ratio 80:20. The spraying methods were described as follows: ninhydrin, iodine vapor and molish reagents for detection of amino acids, lipid domains and carbohydrate compounds, respectively (17, 18).

In addition to these spraying methods, biosurfactant chemical characterization can also be quantitatively determined with some staining assays such as Bradford and Anthrone, were performed for cell-free supernatant. Bradford assay is special to detect and quantify amino acids and bovine serum albumin was used as calibration standard (5). Anthrone reagent is special to detect and quantify the amount of glycolipid and for preparation of standard curve, a pure sample of glycolipid is used (15).

### Electrospray ionization mass spectra (ESIMS)

Individual rhamnolipid congeners were identified by electrospray ionization tandem mass spectrometry equipped with LCQ quadrupole ion trap mass spectrometer (Finnigan MAT, San Jose, CA, USA). The sample was dissolved in methanol at a concentration between 0.01–0.5 mg/ml and negative ion mass spectra were recorded in the *m*/*z* range of 50–1200. This experiment was carried out under the following conditions: syringe 5 μl/ml, nitrogen sheath gas and auxiliary gas at 20 and 35 (arbitrary units), respectively, spray voltage to 4.5 kV, capillary temperature at 250 °C, capillary voltage to 47 V and tune lens offset to 40 V (15).

### Statistical Analysis

Optimization experiments were carried out in triplicates and repeated measures ANOVA analysis was utilized to determine significant differences among carbon sources and C/N ratios (SPSS, ver. 19.0 (Chicago, IL, USA)). The result was considered significant if *P*<0.05.

## Results

### Screening of biosurfactant-producing micro-organisms

After isolation of purified bacterial colonies, they were inoculated in MSM supplemented with 1% (v/v) crude oil. Finally, the best crude oil degrader and emulsifier were selected. The appearance of this colony was wet, convex and also some morphological and biochemical properties were: gram-negative, motive, rod-shaped, results of catalase, oxidase, nitrite reduction, and denitrification tests were positive. 16S rDNA gene sequences indicated that this strain was a species of *Pseudomonas aeruginosa* sp. ZN with an accession number KX808675 (The data was not shown).

### The effects of different carbon sources and C/N ratios on biosurfactant production

**Fig. 1:**
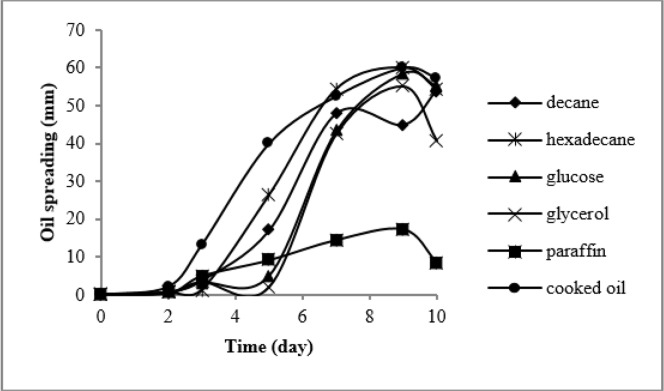
Effect of different carbon sources on biosurfactant production.

Oil spreading curve showed that cooked oil and n-hexadecane were the best carbon sources for biosurfactant production as a result of the maximum diameter of clear zone and the shortest lag phase, 2 and 3 d, respectively. While strain ZN could not use paraffin to biosurfactant production (Fig. 1).

Moreover, cooked oil and n-hexadecane had major effect on biosurfactants production because there was a significant difference between them and other hydrocarbons. E24 test showed that the produced biosurfactants in the presence of cooking oil and n-hexadecane were able to form stable and thick layer of emulsions, approximately 65%.

**Fig. 2:**
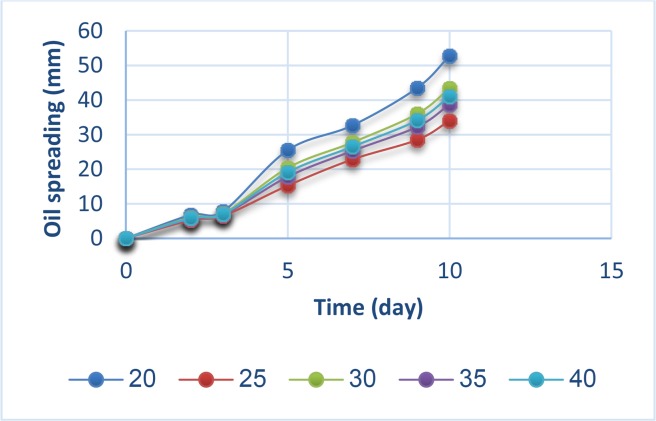
Effect of different C/N ratios on biosurfactant production.

In the case of different C/N ratios, pattern of biosurfactant production was the same for all tested ratios; initially slower in the first 3 days and then with higher rates of growth for all with no significant differences between tested C/N ratios (Fig. 2).

### Kinetic of biosurfactant production

**Fig. 3:**
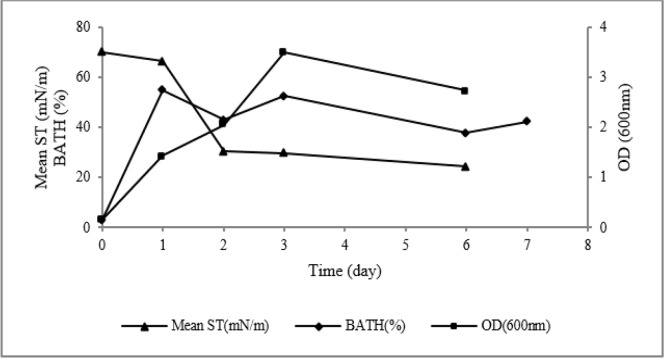
Kinetic of biosurfactant production by *P. aeruginosa* sp. ZN in the modified BH2 medium containing 1 % (w/v) n-hexadecane and BATH test

In the modified BH2 medium containing with 1% (w/v) n-hexadecane, *P. aeruginosa* sp. ZN grew to 3.5 during 3 d; hence, surface tension of cell-free supernatant decreased from 70 to 29 mN/m as its minimum level. Cell hydrophobicity (BATH test or CH %) increased immediately after incubation to maximum value (about 55%); Then the end of incubation time it gradually decreased to approximately 40% (Fig. 3). Although the strain reduced surface tension to about 29 mN/m after 3 d, its CMD was higher compared with what was observed after 6 d.

### Physico-chemical properties of biosurfactant

The produced biosurfactants had no antimicrobial activity against *E. coli* and *C. albicans* while showed weak antibacterial activity against *B. subtilis.*

A mixture of span-80-kerosene: Tween-80-distilled water with ratio 30:70 produced the most stable emulsion after 24 h and was selected as model emulsion. Results from demulsifying activity showed that the biosurfactants could not break the model emulsion after 24 h at room temperature.

### Structural characterization of biosurfactant

**Fig. 4:**
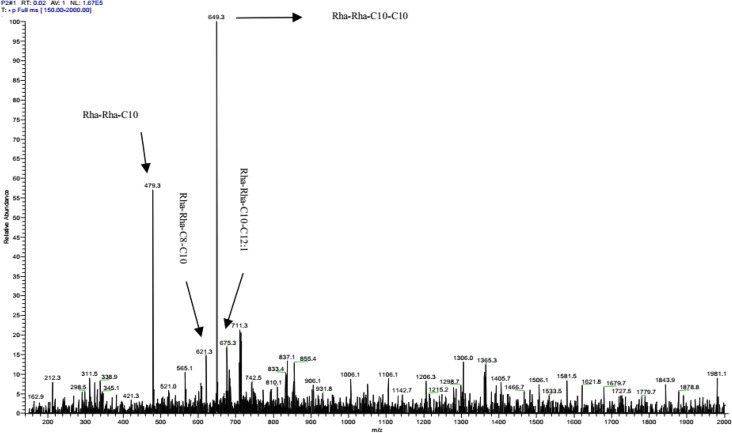
ESI-MS chromatogram of biosurfactant produced by *P. aeruginosa* sp. ZN

Results of preliminary tests of developed TLC spraying assay with ninhydrin, iodine vapor and molish revealed that the biosurfactants produced by this isolate contained three different spots (13, 57 and 65 mm) with a low amount of amino acids, which may be due to presence of residual cell debris, a large amount of carbohydrate and lipid groups. These results were confirmed with staining assays on cell-free supernatant with Bradford and anthrone reagents that showed a large number of glycolipids. The exact characterization result obtained with ESI-MS was indicative that the presence of four different congeners of rhamnolipid: Rha-Rha-C10 (479 *m*/*z*), Rha-Rha-C8-C10 (621 *m*/*z*), Rha-Rha-C10-C10 (649 *m*/*z*) and Rha-Rha-C10-C12:1 (675 *m*/*z*) (Fig. 4).

## Discussion

Strain ZN was selected as high-efficiency biosurfactant producing strain through preliminary screening methods and 16S rDNA sequencing result showed isolated strain exhibited the highest similarity (99%) to *P. aeruginosa*. In the presence of n-hexadecane as carbon source, biosurfactant production occurred on the exponential phase. The result was reported when *P. aeruginosa* MR01 grew in the presence of glucose. Although some biosurfactants such as surfactin were considered as a primarily products and produced during the exponential phase, limitation of some essential medium ingredients especially nitrogen sources, played a key role for biosurfactant production (5, 17, 19). The maximum rhamnolipids production was reported by *P. aeruginosa* UKMP 14T in the presence of glycerol and ammonium sulfate as carbon and nitrogen sources with C/N ratio 14:1 (20). The best C/N ratios for rhamnolipids production were 20:1 and 10:1, respectively, were also confirmed these issue (6, 21). While in the current study, results of oil spreading assay and statistical analysis showed that strain ZN was able to produce biosurfactants in a wide range of C/N ratio between from 10 to 40.

In the medium culture contained n-hexadecane, produced biosurfactant caused increase cell hydrophobicity up to 55%. Production of biosurfactants probably released lipopolysaccharides (LPS) on cell surface of bacterium and increased the cell hydrophobicity (22).

According to amphiphilic properties, some biosurfactants are able to break stable emulsions of oil in water or water in oil (23). In addition, for first time the use of rhamnolipid for demulsification of waste crude oil was cited with efficiency of 98% (24). However, the current study showed that this biosurfactant could not destabilize the model emulsion prepared with span-80-kerosene: Tween-80-distilled water with 30:70 ratios. Some effective factors were reported such as culture age, pH and temperature on demulsifying activity. Therefore, pH and temperature with effects on the ionization of emulsion ingredients or any ionized group on the bacterial surfaces and the viscosity of oil phases, respectively, could be effective on demulsifying activity (25–27).

Many species of *Pseudomonas* produce rhamnolipid but there are some reports of lipopeptides produced by *P. fluorescens* BD5 and *P. putida* (28, 29). Acid precipitation is commonly used to purify glycolipid (rhamnolipid) while for purifying lipopeptide, solvent extraction is frequently used but some authors used acid precipitation as well (30, 31). Ammonium sulfate precipitation is used to purify the majority of high molecular weight biosurfactants with a considerable amount of protein contents (16). However, a very few articles used this method to purify rhamnolipids. In this method after centrifugation, floating materials contained high concentration of biosurfactants (14). The result of preliminary characterizations showed that produced biosurfactants contained some hydrocarbon and lipid compounds and suggested it was glycolipid as expected from species of *P. aeruginosa.* Moreover, the result of ESI-MS confirmed rhamnolipid production with this isolate and it showed 4 different congeners of rhamnolipids (Rha-Rha-C10, Rha-Rha-C8-C10, Rha-Rha-C10-C10 and Rha-Rha-C10-C12:1) in the partially purified biosurfactant.

## Conclusion

*P. aeruginosa* sp. ZN produced rhamnolipids during exponential phase and a wide range of C/N ratios between 10 and 40 had no significant effect on production process. On the other hand, the ability of biosurfactant production in the presence of cooked oil and n-hexadecane is indicative that the using of this strain in order to biodegradation of light fractions of crude oil and wastes of food industries has been optimistic.

## Ethical considerations

Ethical issues (Including plagiarism, informed consent, misconduct, data fabrication and/or falsification, double publication and/or submission, redundancy, etc.) have been completely observed by the authors.
